# Effect of ligustilide on Ang II-induced hypertrophy in cardiomyocytes and the potential mechanisms

**DOI:** 10.3892/etm.2014.1690

**Published:** 2014-04-24

**Authors:** QUN LU, SHAOHONG LUO, YONGFANG WEN

**Affiliations:** Department of Biochemical and Molecular Biology, School of Basic Courses, Guangdong Phamaceutical University, Guangzhou, Guangdong 510006, P.R. China

**Keywords:** ligustilide, angiotensin II, myocardial cells, hypertrophy, apoptosis

## Abstract

The aim of the present study was to investigate the effect of ligustilide (LIG) on angiotensin II (Ang II)-induced hypertrophy in neonatal rat myocardial cells and the expression levels of p53, Bcl-2 and Bax. Myocardial cells were isolated and purified from the ventricles of neonate Sprague-Dawley rats (age, 1–3 days) using a differential adhesion method. The cells were then were stimulated by Ang II and LIG for 1–3 days, following which the cell surface area, intracellular protein concentration, rate of apoptosis and the expression levels of p53, Bcl-2 and Bax were determined. Following stimulation with Ang II, the cell surface area of the neonatal rat myocardial cells increased significantly and the cell morphology was distorted. LIG was shown to significantly suppress the Ang II-induced hypertrophy of neonatal rat myocardial cells in a dose-dependent manner. In addition, administration of LIG restored the expression levels of p53, Bcl-2 and Bax. Therefore, LIG can prevent the hypertrophy of cardiomyocytes induced by Ang II, which may be associated with the inhibitory effect that LIG exhibits on cardiomyocyte apoptosis.

## Introduction

Cardiovascular disease is a common and frequently encountered disease, with the highest morbidity and mortality rates worldwide. Thus, treatment for cardiovascular disease has become an increasingly important not only for clinicians, but also for the pharmaceutical industry. Ligustilide (LIG) has been reported to have a number of biological activities, including anti-spasm, alleviating pain and relieving asthma functions (1). LIG can inhibit the metabolism of platelet arachidonic acid, preventing platelet aggregation (2). LIG can also reduce vascular resistance, increase blood flow and improve microcirculation (3,4). Furthermore, LIG has been shown to exhibit antioxidant effects, thus, can antagonize free radical-induced tissue damage (5). In addition, LIG has been demonstrated to exhibit an inhibitory effect on vascular cell proliferation (6,7). In a previous study, LIG was found to significantly inhibit vascular smooth muscle cell proliferation via the mitogen-activated protein kinase/cycle proteins (p21, CyclinD1 and pRb) and extracellular signal-regulated kinase signaling pathway. Thus, LIG also exhibits certain therapeutic effects in cardiovascular diseases (8). The proliferation and migration of vascular smooth muscle cells is a key process in the formation of atherosclerosis. In addition, cardiac hypertrophy and fibrosis results in diseases, including systolic rhythm imbalance. In this respect, the present study analyzed whether LIG exhibits a similar therapeutic effect in heart disease, such as the proliferation, migration and invasion of vascular smooth muscle cells, and the cardiac hypertrophy. LIG may have a large clinical value if it does have an inhibitory effect on cardiac hypertrophy. Thus, in the present study, the effects of LIG on angiotensin II (Ang II)-induced hypertrophy of myocardial cells was preliminarily investigated, as well as the possible underlying mechanisms, in order to provide a scientific basis for the development of a novel drug for the treatment of cardiovascular disease.

## Materials and methods

### Animals and reagents

Sprague-Dawley (SD) neonatal rats (age, 1–3 days) were provided by Guangzhou University of Chinese Medicine (Guangzhou, China), the study was approved by the ethics committee of Guangdong Phamaceutical University, (Guangzhou, China). Animals were treated according to the animal care guidelines of Guangdong Pharmaceutical University. Ang II was purchased from Alexis Corporation (Leistal, Switzerland) and ligustilide was purchased from Tianjin Hualida Biotechnology Co., Ltd. (Tianjin, China). Fetal bovine serum (FBS) was obtained from Zhejiang Tianhang Biological Technology Co., Ltd. (Hangzhou, China) and Dulbecco’s modified Eagle’s medium (DMEM) was purchased from Invitrogen Life Technologies (Carlsbad, CA, USA). Primary antibodies against p53, Bax and Bcl-2, as well as a secondary antibody, were purchased from Wuhan Boster Bioengineering Co., Ltd. (Wuhan, China). A cytometric bead array (CBA) assay kit and 3,3′-diaminobenzidine (DAB) kit were purchased from the Beyotime Institute of Biotechnology (Shanghai, China).

### Preparation of primary myocardial cells

Cardiac ventricles from the neonatal SD rats were removed under sterile conditions and placed into pre-cooled D-Hanks’ medium. Following cutting into sections and digestion with 0.125% trypsin, the cell suspension was filtered and centrifuged at 1,000 × g for 10 min. The cells were then resuspended in DMEM containing 20% FBS and incubated in an atmosphere of 37°C and 5% CO_2_ for 90 min to allow for cell adherence. Next, the supernatant containing the inadherent cells was harvested and cultured with 0.1 mmol/l bromodeoxyuridine for 24 h to suppress the proliferation of non-myocardial cells. The medium was replaced with serum-free DMEM and incubated in an incubator with saturated humidity and 5% CO_2_ at 37°C. Following incubation for 24 h, the myocardial cells were prepared for the following stimulation experiment.

### Treatment

Prepared myocardial cells were divided into three groups and treated with various stimuli. The control group was cultured normally. The Ang II group was treated with 1 μg/ml Ang II, while the Ang II + LIG group was subdivided into three subgroups and treated with 1 μg/ml Ang II and various doses of LIG (25, 50 and 100 μg/ml). Each group and subgroup was incubated in an incubator with saturated humidity and 5% CO_2_ at 37°C for one to three days. The cell surface area, total protein content, apoptosis rate and the expression levels of p53, Bax and Bcl-2 of the cultured cells were then detected.

### Effects of LIG on the hypertrophy of cardiomyocytes

Following incubation for one to three days with various stimuli, the cells were observed under an inverted microscope.

### Detection of the myocardial cell surface area

Following incubation for two days with various stimuli, cells were stained with hematoxylin and eosin and observed under an inverted microscope. The myocardial cell surface area was detected using an Image-Pro Plus image analysis system. Five fields were selected randomly for detection and between 10 and 15 cells in each field were randomly selected and measured. Each group measurements were performed in triplicate.

### Determination of the cellular total protein concentration

Following incubation for two days with various stimuli, the cells were harvested and washed with phosphate-buffered saline (PBS) three times. Cells were then lysed with radioimmunoprecipitation assay buffer for 10 min and centrifuged at 13,400 × g at 4°C for 3 min. The supernatant was collected and the total protein content was determined with a CBA determination assay kit.

### Determination of the apoptotic rate

Following incubation for two days with various stimuli, the cells were harvested and rinsed twice with PBS. The cell concentration was adjusted to 1×10^5^ cells/ml, and 100 μl cell suspension was mixed with Tris-HCl buffer (containing 1% RNase) and incubated for 10 min. Next, 5 μl annexin and 5 μl propidium iodide (PI) were added to the cell suspension. Following incubation at 37°C for 30 min in dark, the apoptotic rate was detected using flow cytometry (FACS Calibur cytometer; BD Biosciences, Franklin Lakes, NJ, USA).

### Immunocytochemistry analysis

Following incubation for one day with various stimuli, the cells cultured on the coverslips were fixed with 4% paraformaldehyde for 30 min, which was followed by incubation with 3% H_2_O_2_ (H_2_O_2_:methanol, 1:50) at room temperature for 20 min to block endogenous peroxidase. Following washing with distilled water and soaking with PBS, the cells were incubated with 5% bovine serum albumin at 37°C for 30 min. The cells were then incubated with anti-p53, anti-Bax or anti-Bcl-2 antibodies at 4°C overnight. After washing with PBS three times, the coverslips were incubated with a secondary polymeric, peroxidase-labeled antibody for 1 h. The positive signals were detected with a DAB kit and observed under a light microscope.

### Statistical analysis

Statistical analysis was conducted with three or more groups using one-way analysis of variance and Dunnett’s test. Data are expressed as the mean ± standard deviation and P<0.05 was considered to indicate a statistically significant difference. SAS System for Elementary Statistical Analysis was provied by SAS company (Cary, NC, USA).

## Results

### LIG restores Ang II-induced hypertrophy of myocardial cells

**Figure 1:**
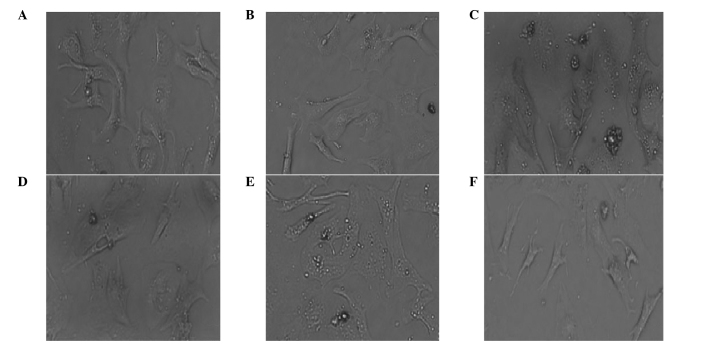
Effects of LIG on the hypertrophy of cardiomyocytes (magnification, ×200) in the (A) control at 2 days, (B) control at 3 days, (C) Ang II (1 μg/ml) at 2 days, (D) Ang II (1 μg/ml) at 3 days, (E) Ang II (1 μg/ml) + LIG (100 μg/ml) at 2 days and (F) Ang II (1 μg/ml) + LIG (100 μg/ml) at 3 days. LIG, ligustilide; Ang II, angiotensin II.

Myocardial cells isolated from neonatal SD rats were cultured normally or treated with Ang II and/or LIG. Control myocardial cells grew adherently and extended their pseudopodia normally. Following two days of culture, the cells became triangular or polygon-shaped and a few single cells even beat spontaneously. Pseudopodia were further interwoven into a network and gradually formed clusters or a monolayer, which appeared radiate in concentric circles. Cells pulsed in synchronicity with complete morphology and good vitality (Fig. 1A and B). However, the cells treated with Ang II exhibited evident distortion and fusion, and the cell surface area increased significantly, indicating that hypertrophy occurred in the primary myocardial cells following treatment with Ang II. Following incubation with Ang II for two or three days, the cells became over-hypertrophic. No clear interval between the cells was observed and the fine pseudopodia were integrated into the intercellular space (Fig. 1C and D). Cells also lost their spontaneous beating ability. However, with regard to the cells treated with 1 μg/ml Ang II and 100 μg/ml LIG for three days, the hypertrophic cells restored their original state of normal myocardial cells (Fig. 1E and F).

### LIG reduces the Ang II-induced protein content increase in the myocardial cells in a dose-dependent manner

**Figure 2:**
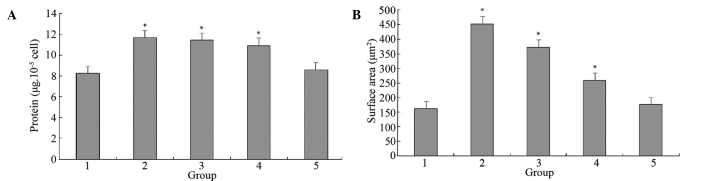
Effects of LIG on the (A) intracellular protein content and (B) cell surface area. ^*^P<0.05, vs. control. 1, control; 2, Ang II (1 μg/ml); 3, Ang II (1 μg/ml) + LIG (25 μg/ml); 4, Ang II (1 μg/ml) + LIG (50 μg/ml); 5, Ang II (1 μg/ml) + LIG (100 μg/ml); LIG, ligustilide; Ang II, angiotensin II.

The total protein concentration was determined in the myocardial cells of the various groups. The total protein content of the cells treated with Ang II for two days increased markedly, however, this increase was significantly reduced with the administration of LIG. Various doses of LIG (25, 50 and 100 μg/ml) exhibited different inhibitory effects on the hypertrophy and protein increase induced by Ang II, with inhibitory rates of 18, 43 and 60% for hypertrophy (P<0.05) and 4, 11 and 27% for protein increment (P<0.05), respectively (Fig. 2). The protein content and cell surface area of the cells treated with Ang II and 100 μg/ml LIG were not significantly different from those of the control group (P>0.05), indicating that LIG can effectively inhibit the Ang II-induced increase in protein synthesis and cell surface area in myocardial cells.

### LIG inhibits the Ang II-induced apoptosis of myocardial cells

**Figure 3:**
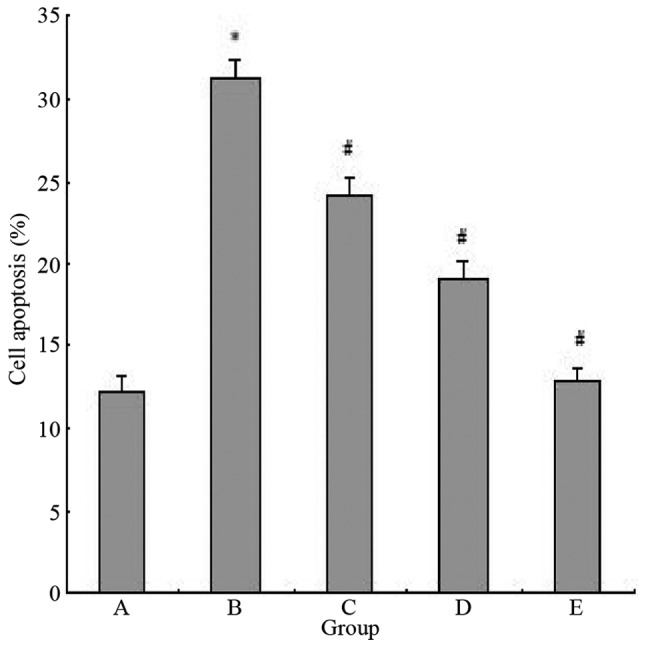
Effects of LIG on the cardiomyocyte apoptotic rate. A, control; B, Ang II (1 μg/ml); C, Ang II (1 μg/ml) + LIG (25 μg/ml); D, Ang II (1 μg/ml) + LIG (50 μg/ml); E, Ang II (1 μg/ml) + LIG (100 μg/ml) ^*^P<0.05, vs. control; ^#^P<0.05, vs. Ang II group B. LIG, ligustilide; Ang II, angiotensin II.

In order to determine whether LIG also affects the Ang II-induced apoptosis of myocardial cells, the apoptotic rates of cells treated with Ang II and/or various doses of LIG were determined. The apoptotic rate of myocardial cells induced by Ang II was markedly higher when compared with the control group (P<0.05). However, the apoptotic rate was significantly reduced with LIG administration (P<0.05; Fig. 3).

### Effect of LIG on the expression levels of the apoptosis-associated proteins, p53, Bcl-2 and Bax

**Figure 4:**
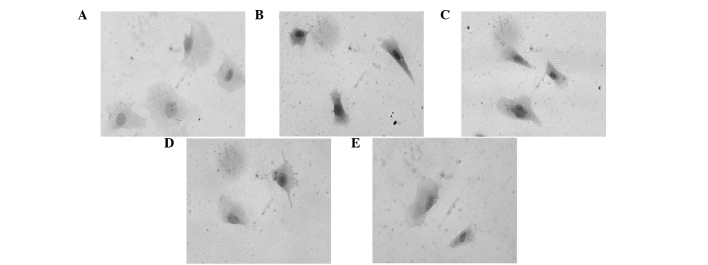
Effects of LIG on the expression levels of p53 in cardiomyocytes (magnification, ×400; DAB & hematoxylin stained) in the (A) control at 1 day, (B) Ang II (1 μg/ml) at 1 day, (C) Ang II (1 μg/ml) + LIG (25 μg/ml) at 1 day, (D) Ang II (1 μg/ml) + LIG (50 μg/ml) at 1 day and (E) Ang II (1 μg/ml) + LIG (100 μg/ml) at 1 day. LIG, ligustilide; Ang II, angiotensin II.

**Figure 5:**
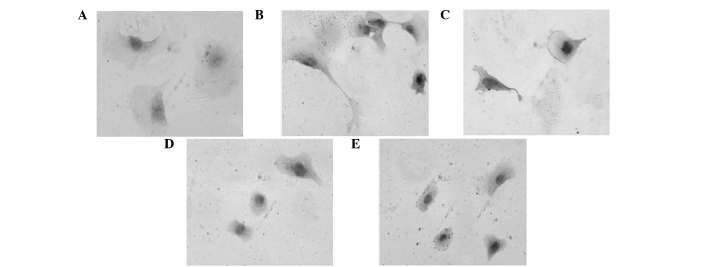
Effects of LIG on the expression levels of Bax in cardiomyocytes (magnification ×400; DAB & hematoxylin stained) in the (A) control at 1 day, (B) Ang II (1 μg/ml) at 1 day, (C) Ang II (1 μg/ml) + LIG (25 μg/ml) at 1 day, (D) Ang II (1 μg/ml) + LIG (50 μg/ml) at 1 day and (E) Ang II (1 μg/ml) + LIG (100 μg/ml) at 1 day. LIG, ligustilide; Ang II, angiotensin II.

**Figure 6:**
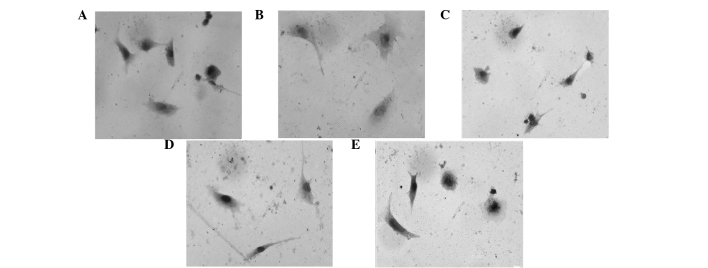
Effects of LIG on the expression levels of Bcl-2 in cardiomyocytes (magnification, ×400; DAB & hematoxylin stained) in the (A) control at 1 day, (B) Ang II (1 μg/ml) at 1 day, (C) Ang II (1 μg/ml) + LIG (25 μg/ml) at 1 day, (D) Ang II (1 μg/ml) + LIG (50 μg/ml) at 1 day and (E) Ang II (1 μg/ml) + LIG (100 μg/ml) at 1 day. LIG, ligustilide; Ang II, angiotensin II.

Expression levels of p53, Bcl-2 and Bax in the primary rat myocardial cells were detected by immunocytochemistry analysis. The expression of p53 and Bax in the myocardial cells was markedly induced with Ang II treatment, which was observed as black staining in the nuclei. However, the expression of these proteins was inhibited with various doses of LIG, with administration of 100 μg/ml LIG restoring the expression levels of p53 and Bax almost to the levels in the normal myocardial cells (Figs. 4 and 5). By contrast, the expression of Bcl-2 was significantly suppressed with Ang II treatment, although this was restored by various doses of LIG. With increasing doses of LIG, the expression levels of Bcl-2 gradually increased, as shown by the black staining (Fig. 6).

## Discussion

It is generally hypothesized that Ang II is a classic inducer of cardiac hypertrophy. The results of the present study demonstrated that the cell surface area and the total protein content of the neonatal rat myocardial cells increased significantly when the cells were incubated with Ang II for 48 h, which was consistent with the results reported by previous studies (9–12). In the current study, an experimental model of cardiac hypertrophy induced by Ang II was successfully established. The observations demonstrated that LIG significantly inhibited the process of cardiac hypertrophy. In addition, LIG inhibited the Ang II-induced increase in cell surface area and protein concentration in myocardial cells in a dose-dependent manner.

Previous studies have demonstrated that the apoptosis and proliferation of myocardial cells occurs in overload-induced ventricular hypertrophy (13,14). Apoptosis, proliferation and hypertrophy are processes that are closely associated with each other, since the developmental stages of the ventricular and are changes in ventricular remodeling. Apoptosis is an initiative cellular suicide process that is regulated by a number of genes, the majority of which are tumor-associated genes, including proto-oncogene and anti-oncogene. Bcl-2, p53 and Bax genes are apoptosis-associated genes. Overexpression of p53 can not only trigger the apoptotic process, but can also inhibit cell cycle progression from the G1 phase to the S phase (14,15). A low expression level of Bcl-2 and a high expression level of Bax promotes the occurrence of apoptosis (16–18).

In China, Suxiao Jiuxin pills and compound Danshen (*Salvia miltiorrhiza*) tablets are widely administered for the treatment of cardiovascular disease. These treatments significantly improve the patient’s clinical symptoms, signs and electrocardiograms. It has been reported that Danshen (19,20) and Chuanxiong (Szechwan Lovage Rhizome) (21,22) exhibit the characteristics of anti-ischemia, anti-hypoxia and calcium channel blockers. These treatments have been shown to block the L-type calcium current in the ventricular myocytes and antagonize Ca^2+^ influx in myocardial cells (23). Previous studies have also demonstrated that Danshen and Chuanxiong have an inhibitory effect on cardiac hypertrophy, as they have been shown to inhibit the formation of left ventricular hypertrophy in spontaneously hypertensive rats (19–22). LIG is one of the active ingredients in Danshen and Chuanxiong. In the present study, LIG was shown to markedly reduce the cell surface area and protein concentration of hypertrophic myocardial cells. The topic investigated the effects of LIG on myocardial cells, the cell surface area, the intracellular protein concentration, the rate of apoptosis and the expression levels of p53, Bcl-2 and Bax were determined. The results demonstrated that LIG reversed the Ang II-induced hypertrophy in cardiomyocytes and restored the expression levels of the apoptosis-associated proteins, p53, Bcl-2 and Bax, indicating that LIG exhibits a significant preventive effect on cardiac hypertrophy. These observations may be associated with the inhibitory effects that LIG exhibits on the apoptosis of myocardial cells. However, the specific underlying mechanism of LIG requires further investigation.
